# Exploring the stigma against people with mental illness in Bangladesh

**DOI:** 10.1017/gmh.2024.107

**Published:** 2024-11-11

**Authors:** Prodyut Roy, Kamal Uddin Ahmed Chowdhury

**Affiliations:** 1 Independent Scholar – Educational Psychology, Dhaka, Bangladesh; 2 Department of Clinical Psychology, University of Dhaka, Dhaka, Bangladesh; 3 Nasirullah Psychotherapy Unit, Department of Clinical Psychology, University of Dhaka, Dhaka, Bangladesh

**Keywords:** Bangladesh, self-stigma, public stigma, professional stigma, institutional stigma

## Abstract

This study investigates the stigma against people with mental illness in Bangladesh through in-depth interviews with 14 patients and 9 healthcare professionals, and 33 focus group discussions with people without mental illness. The research has delved into the understanding of different types of stigma against mental illness in the context of Bangladesh. The findings revealed four types of stigma which were categorized into four themes namely self-stigma, public stigma, professional, and institutional stigma. Patients had internalized negative attitudes, thereby discriminated toward themselves. The public discriminated against patients because of believing in prejudices against them. Other health professionals had negative conceptions toward patients, and they devalued mental health professionals (MHPs). A culture of negative attitude and belief had emerged in institutional settings which encouraged discrimination. Policymakers and healthcare professionals can use the findings to develop a mental health service by addressing the stigma. Mental health practitioners can assess the impact of stigma to improve the mental well-being of their patients. Students and workplace staff will benefit from intentional or unintentional discrimination in educational institutions and workplace settings by addressing the effects of stigma. Importantly, other health care providers will be aware of their thoughts against patients and MHPs.

## Impact statement

There is a significant gap in knowledge about understanding specific types of stigma against mental illness because other studies in Bangladesh have focused on examining the impact of stigma on treatment-seeking behavior and accessibility in getting treatment against mental illness. This study explores different types of stigma against mental illness by recruiting a larger sample that represents the diverse community of the country including marginalized populations (e.g., recovered substance users, Indigenous community, persons with disabilities, transgender community, and adolescents with a single parent or parentless group). The data reflect the experience and viewpoints of patients with mental illness, health care professionals, and general people without mental illness. With the findings, policymakers can address the stigma to improve the health care system. Mental health professionals (MHPs) will know the nature of stigma experienced by their patients that will help them to make an effective and holistic intervention plan to improve encountered psychological problems of their patients. Other non-MHPs will be aware of the existing stigma and its impact on the well-being of their patients. The findings of this study also furnish the literature regarding mental health-related issues in Bangladesh as well as similar cultural and economic countries.

## Introduction

Stigma against mental illness hampers every sphere of life of a patient having a mental illness (Ahmedani, 2011; Corrigan et al., 2014). The term “patient” will be used to mean a patient having a mental illness in this article. According to Goffman (1963), stigma is “an attribute that is deeply discrediting” (p.3). The stigmatization of mental illness refers to humiliation, community condemnation, or any acts that devalues and taints people with a mental illness (Goffman, 1963).

Based on the literature, there are four types of stigma against mental illness namely self-stigma, public stigma, professional stigma, and institutional stigma. Self-stigma is a negative attitude of patients against themselves due to having a mental illness (American Psychiatric Association, 2020; Corrigan et al., 2012; Thornicroft et al., 2022). Public stigma refers to the general people’s negative and discriminatory behaviors toward patients (American Psychiatric Association, 2020; Corrigan et al., 2012). Professional stigma is defined as the healthcare professional’s negative attitude and discriminatory behaviors toward patients. It also includes the negative and prejudiced attitude of patients, general people, or other healthcare professionals toward mental healthcare professionals due to interaction and work with patients who are already stigmatized in the society (Ahmedani, 2011). Finally, institutional stigma is defined as the intentional or unintentional policies of an organization and negative attitudes toward patients that restrict the opportunities for them (Ahmedani, 2011; American Psychiatric Association, 2020; Brohan and Thornicroft, 2010; Corrigan et al., 2012, Corrigan et al., 2014; Thornicroft et al., 2022).

Literature reviews provide evidence that stigma not only affects patients’ lives but also alters the lives of family members, relatives, and mental health professionals (MHPs). Stigma is also considered as a burden for both the personal and social lives of patients (Gaebel et al., 2011). Patients and their family members may refrain themselves from seeking treatment against mental illness; therefore, it increases the severity level of psychological problems they are suffering (Corrigan and Kleinlein, 2005; Corrigan et al., 2012; Corrigan et al., 2014; Rüsch et al., 2014). In addition, research evidence also suggests that mental illness stigma not only develops additional psychological problems for patients but also it weakens support system and community network (Corrigan et al., 2014). In terms of the development of diverse mental illness, we can start with self-efficacy (Kleim et al., 2008) and self-esteem (Link et al., 2001; Corrigan, 2004) which are diminished significantly because of stigma. Stigma also plays a crucial role in increasing psychological symptoms by declining coping mechanisms of patients (Stevens et al., 2009; Verhaeghe et al., 2010). Moreover, individuals can develop physical diseases such as back pain and obesity because of stigma (Sickel et al., 2014). It may also affect the treatment of physical diseases for a patient having a psychological problem (Livingston, 2013).

However, stigma against mental illness in South-Asian countries is prominent and affects health-seeking behavior and quality of treatment for mental illness. For example, in Afghanistan, the word “mental illness” was highly stigmatizing (Nine et al., 2022). Due to mental illness-related stigma, the quality of care for patients was seriously hampered in Bhutan (Pelzang, 2012). Mental illness-related stigma was considered as one of the barriers to access treatment for mental illness in Nepal (Petersen et al., 2017) whereas treatment for psychological problems was provided by shamans in Pakistan (Waqas et al., 2014).

Kaur et al. (2021) conducted a systematic review to understand interventions for reducing stigma in India. They found only 9 articles about interventions for breaking down stigma from 1990 to 2020. In another study, Kaur et al. (2023) found that people from Northern India had a lack of awareness and knowledge about mental health. They also found that a dire gap in the implementation of mental health policies existed for the provision of mental health services among people. However, in recent years, intensive initiatives have been taken to reduce stigma in India. For instance, SMART (Systematic Medical Appraisal, Referral and Treatment) mental health project (Maulik et al., 2019), ARTEMIS (Adolescents’ Resilience and Treatment nEeds for Mental health in Indian Slums) intervention research project (Yatirajula et al., 2022) are noticeable initiatives within the country. Moreover, The Indigo Partnership research project is one of the initiatives to understand the underlying mechanisms of stigma manifestation in China, Ethiopia, India, Nepal, and Tunisia (Gronholm et al., 2023). Gurung et al. (2023) designed an intervention named RESHAPE (REducing Stigma among HealthcAre ProvidErs) to reduce stigma among primary care providers in these countries.

Bangladesh is a Southeast Asian country where stigma against patients has engulfed the entire country including mental health treatment services (Hasan and Thornicroft, 2018). Here in Bangladesh, patients and family members encounter discrimination because of having a patient in their family likely the other developing countries in the world (Soron, 2015). Autism is also considered as a mental illness, and parents become victims of social stigma (Soron, 2015).

National Institute of Mental Health (2021), a government mental health hospital in Bangladesh, in their recent national survey reported a higher prevalence (37.6% to 98.2%) of stigma toward mental illness where men perceived a higher level of stigma in comparison to the women population. Patients experienced similar level of discriminations irrespective of their geographical locations in Bangladesh. Faruk et al. (2023) conducted a study and found predictive factors of stigma namely age, gender, socioeconomic status, treatment-seeking behavior, and knowledge about mental health. Females experienced more stigma against mental illness than males in the sub-scale (i.e., anxiety, hygiene, and relationship disruption) of the mental illness stigma scale developed by Day et al. (2007). Females sought treatment for their mental illness more frequently in comparison to male. Their study results also indicated that experiencing stigma increased with the increasing of age. For example, respondents aged more than 40 years old experienced more stigma in the sub-scale of anxiety and relationship difficulties than participants aged between 40 and 25 years old. From the socioeconomic status (SES) perspective, respondents of the middle class and lower-middle class had more stigma than people of lower SES. They also found that individuals with knowledge of mental health had lower stigma against mental illness in comparison to individuals without having knowledge of mental health. Moreover, they also found a high prevalence of stigma that supported the study of National Institute of Mental Health (2021) in Bangladesh.

### Research gap, significance, and question guiding this inquiry

Literature review indicates that most of the research focused on examining the impact of stigma. For example, Islam et al. (2008) found a negative correlation between stigma and treatment seeking behavior against mental illness. In another research, Nuri et al. (2018) found stigma as one of the responsible barriers to access mental health services. A significant research gap exists in the understanding of specific types of stigma in this context. Hence, it is important to understand the different types of stigma that are embedded in society in reducing the impact of stigma on both the life of patients, family members, and mental health care professionals. The present study uses qualitative approach to explore different types of stigma which grants us to gain a comprehensive insight into stigma in the context of Bangladesh. This insight will help mental health practitioners to assess the impact of stigma and to formulate intervention in improving the mental well-being of their patients. Policymakers of the country are better able to understand the types and impact of stigma. Moreover, the findings of this study can offer researchers, policymakers, and health professionals (HPs) even general people including patients a deeper understanding of stigma through the lens of wider Asian cultural context.

## Methodology

### Research design

A case study method was employed to reveal mental illness related stigma in Bangladesh. The case study method offers a complete understanding of a topic because it “is an empirical enquiry that investigates a contemporary phenomenon within its real-life context” (Yin, 1994). In addition, this method admits researchers to collect data from different stakeholders (Yin, 2009) .

### Study setting and participants

This study was conducted in Bangladesh covering all eight divisions of the country such as Barishal, Chattogram, Dhaka, Khulna, Rajshahi, Rangpur, Mymensingh, and Sylhet. Adolescents (age ranges 12 to 17 years) and adults (18 years and above) people in the country were the population for the present study. Purposive sampling technique was used to recruit respondents because this technique is very effective and used widely in qualitative inquiry (Patton, 2002). Participants were (1) patients, (2) people without mental illness, and (3) HPs of government and private health care facilities in Bangladesh. The literature review indicated a higher level of stigma against mental illness and patients in the community; therefore, selecting respondents randomly was quite impossible because of stigma. Here, purposive sampling solved this challenge to recruit participants.

Data were collected through in-depth interviews with patients and healthcare professionals and focus group discussions (FGD) with people without mental illness. Saturation was the criterion to determine the sample size for qualitative inquiry (Miles and Huberman, 1994; Mason, 2010). Participants for in-depth interviews were 14 patients (both male and female from adolescent and adult) and 9 healthcare professionals (e.g., general physician, psychiatrist, psychologist, and nurse). Thirty-three FGDs consisted of 193 people without mental illness were recruited. A diverse population based on their jobs, ethnicity, gender identity (e.g., transgender), disability, and having single parents or parentless were identified and selected for the study. Homogeneity was followed to form a group. For instance, a group was formed with Indigenous people whereas another FGD was conducted with a group of persons with disabilities. Characteristics of participants of in-depth interviews are presented in Supplementary Tables 1 and 2, and number and categories of the FGD respondents are provided in Supplementary Table 3. A short description of participated groups in the FGD would help readers to understand them. For the group of “substance abuse,” participants had a previous history of substance abuse, and all were admitted in drug rehabilitation centers. They recovered and led a normal life during the conduction of FGD. For group of “Indigenous community,” participants were from different Indigenous communities such as Manipuri, Chakma, Tanchangya, and Assam. “Housewife” referred to homemakers who were not working (i.e., income-generating activities) with organizations. They performed household activities such as cooking, washing, and household related tasks for their family. “Working women” referred to participants who were involved in income-generating activities with different organizations. “Teachers” were also doing income generating activities with organizations. The difference between the category group of “working-women” and “teachers” was the working-women category consisted of different professionals from various fields, while the teacher category was composed solely of individuals in teaching roles. “Having single parent/parentless adolescents” group indicated that most of the participants of these groups were underprivileged adolescents. Some of them had single parents and some of them had no parents. “Community” were members of the community, but it was difficult to differentiate them by their education and job. For example, the community group in Dhaka division consisted of students and workers. “Student” were respondents from different schools and colleges where madrasa students were from Islamic religious-based academic institutions. For “PWD” group, all participants had vision impairments. Therefore, they were able to make meaningful communication using audio sound.

Patients were selected from psychiatric departments of hospitals where they were diagnosed by psychiatrists. Here, patients who were unable to make meaningful communication with people were excluded from the study. For example, patients with a diagnosis of schizophrenia and did not have insight into reality were excluded from the study. For selecting of people without mental illness, pre-screening questions were asked to them. Research assistants for this study were graduates of the Department of Clinical Psychology at the University of Dhaka. They conducted a short interview with potential participants. For instance, respondents were asked about their psychiatric history such as whether they were taking any psychiatric medications, counseling services or not. In addition, participants declared by themselves that they were not experiencing any severe psychological problems that demanded consulting a mental health care professional.

### Data collection procedures

An approval from the ethics committee of the Department of Clinical Psychology, University of Dhaka was taken before data collection. After that approval was granted from healthcare facilities from where data was collected. Research objectives, procedures, roles, and responsibility of participants were explained to all respondents. A written consent was taken from them for data recording (audio) and participation in the research. For adolescent participants, informed consent was taken from their parents or guardians.

FGD and in-depth interviews were the primary method of data collection. In-depth interviews were conducted with patients and healthcare professionals because of several reasons. Firstly, patients shared that they would not feel comfortable with other patients. Secondly, gathering several patients at a time was very challenging due to unavailability. Finally, for healthcare providers, data was collected during working time. Organizing a group with several healthcare professionals from a hospital would interrupt the delivery of health services of hospitals. Considering these challenges, we conducted in-depth interviews with patients and healthcare professionals. Furthermore, FGD was conducted in community settings where mass people participated in the group discussions. The purpose of recruiting participants based on their jobs, ethnicity, disabilities, and other categories was to explore mental illness related stigma in diverse perspectives. For instance, people from an Indigenous community or transgender community may reveal an additional dimension through the lens of their experience against mental illness related stigma.

The FGD and in-depth interviews were conducted using semi-structured interview guides which were designed separately for each category of respondent. The guides were developed based on the literature review. The following two questions were the same for all respondents, though probing questions were tailored to specific participant group.

1. Could you share your opinion about how people with mental illness are treated in Bangladeshi society?
2. Could you please share your experience about living in Bangladesh who have a mental illness?

Under each question, there were several probing questions. The probing questions, for example, patients were asked to share their experience of how other people (e.g., their family members, neighbors, relatives, colleagues, healthcare professionals) treat them because of having a mental illness. For MHPs, an additional question was asked where their experience of working in a mental health hospital was explored. On contrary, patients and people without mental illness were asked to share their experience of stigmatizing attitude from healthcare professionals. Here, patients were asked to share their experience as a patient whereas people without mental illness shared their witnessed discriminatory attitudes against patients.

Eight graduates from the Department of Clinical Psychology at the University of Dhaka were recruited as research assistants to conduct FGD and in-depth interviews from eight divisions of the country. One research assistant was assigned to one division for data collection. Before the conduction of FGD and in-depth interview session, 10 h training was provided to them. We collected data from participants in two phases. The first phase of data collection and writing transcripts took place from 5 March 2022 to 31 March 2022. During this tenure, we conducted 50 sessions (30 FGDs and 20 in-depth interview sessions). In the second phase of data collection, we conducted six sessions (three in-depth interviews and three FGDs) which took place from 1 April 2022 to 16 May 2022. The purpose of the second phase of data collection was to strengthen the credibility of the research findings by examining the theoretical saturation of the research findings. Thirty-one FGDs and 22 in-depth interview sessions were face-to-face sessions. Due to Ramadan and school examination, we experienced challenges to organize sessions based on mutual time and place. Hence, two FGDs and one in-depth interview session were conducted using an online platform.

FGD and in-depth interview sessions took place in safe locations where privacy and confidentiality were maintained. It took on average 1 h (approximately) to conduct FGD sessions whereas 50 min (on average) was needed to conduct an in-depth interview session. We conducted FGD and in-depth interview sessions in native language of Bangladesh (Bengali). For reporting purposes, we translated the selected quotations into the English language.

### Data analysis

We employed deductive (directed) content and inductive analysis approach to analyze the data using ATLAS.ti 22, a qualitative data analysis application. Content analysis is widely used data analysis tool (Hsieh and Shannon, 2005; Bengtsson, 2016) while inductive analysis offers a bottom-up approach in providing a comprehensive understanding of the experiences of respondents (Saldaña, 2013). Content analysis served the research purpose of this study because we aimed to reveal mental illness related stigma in Bangladesh according to the categories of stigma against mental illness based on the literature. In addition, we aimed to acquire an innate understanding of the experiences of participants that was served by inductive analysis.

Interview transcripts were read several times to get an insight from the data. After that, line by line coding approach was followed for generating initial codes. These codes were categorized, and these categories were organized into themes which were based on the classification of stigma against mental illness.

### Rigor and trustworthiness

The researchers needed to employ and embedded techniques in the research process to establish rigor and trustworthiness from beginning (Cypress, 2017). We applied reflexivity and triangulation of data techniques to achieve rigor and trustworthiness of the study since at least two techniques were recommended by Creswell and Poth (2018) to use in establishing rigor and trustworthiness from nine techniques which was adopted from Creswell and Miller (2000).

For reflexivity and bracketing purposes, the first author of this study designed and analyzed the data; therefore, he examined his subjectivity, values, and biases. Then he maintained a reflective journal. For example, he bracketed his experience that was gained from consulting patients. He developed semi-structured interview guides based on the literature, not applying subjective and experience from clinical practice. Moreover, initial coding and categories from initial coding were conducted based on the interview transcripts.

For triangulation of data, different sources of data such as data from FGDs and in-depth interviews were used to triangulate the data for the present study. For instance, patients shared their experiences of discrimination in their family, community, and workplace. Data from healthcare professionals, and people without mental illness supported these findings through cross-checking the results through the lens of experience of different categories of participants.

## Results

We organized findings into four themes (i.e., four types of stigma against mental illness). The findings provide insights about prejudices, discriminatory attitude against mental illness and patients. The findings are presented in Supplementary Table 4.

### Theme 1: self-stigma

Categories of self-stigma theme were generated from the narratives of patients. They expressed their perceived prejudice against mental illness as well as the discriminatory behavior conducted toward themselves because of having a mental illness and prejudice against mental illness.

#### Internalized negative attitude

Patients had internalized negative attitude against their psychological problems. They perceived that their lifestyle was messy due to the lack of self-care and sleep. Their speech would be irrelevant. For example, an adult male patient said, “patient’s dress remains messy. Their dressing style will not be as like as normal healthy people. If they were well, they could comb their hair. But hair will be messy when they will be sick.”

Patients experienced that their family members would treat mental illness as an excuse. After sharing psychological problems they were experiencing, the family members would treat the psychological problems as the method of fulfilling patients’ desires. For instance, an adult male patient stated that if he shared his psychological problems with his family members, his family members would think that he wanted to marry. On contrary, after diagnosed of having a mental illness, patients found difficulties to get married. An adult patient said, “getting married becomes a real challenge. Or it becomes impossible to arrange a marriage later.” They believed that getting married would be impossible because of their psychological problems.

Furthermore, they felt that they would be a burden to their family and be treated as inferior. They felt that family members were ashamed of having them as a family member. One participant reported:

Family is ashamed of having a patient and often face embarrassing situation because of him. They are so ashamed of having him that they cannot present him to society. (Adult male patient)

In addition, nobody wanted to mingle with patients; thus, they were not invited to any social gatherings. Consequently, they believed that they became dangerous person because of their mental illness.

#### Discrimination toward self

This section focuses on the discriminatory behavior that was committed by patients toward themselves. When patients experienced discrimination from their family, society, workplace, and educational settings; they started to commit discriminatory behavior toward themselves. For example, they kept them isolated from family and society. An adult male patient said, “you see the pain I am going through; I am keeping myself far away from all my relatives and friends.” Furthermore, they shared that their tendency to self-care was reduced considerably.

### Theme 2: public stigma

Categories of public stigma were derived from all group of participants, though mostly from the narratives of respondents from FGDs. Patients and general physicians were members of the community; therefore, they had prejudices against patients as well as witnessing discrimination toward patients. Hence, public stigma theme will be elucidated by the viewpoints of all respondents.

#### Prejudice against patients

General people treated patients as a source of fear. They were afraid because they assumed that (1) patients meant aggressive and they could make deadly attacks, (2) they beat people, (3) patients as a host of evil power, and (4) they would create embarrassing situation. A general physician said, “these people are literally schizophrenic patients. …but this people, sometimes, they become violent. These people are dangerously violent.” An adolescent male participant from FGD with students stated that if patients became aggressive, then they could attack.

Furthermore, people were frightened because they presumed mental illness as contagious. They believed that children got mental illness from mother having mental illness. Therefore, they kept patients hidden from both community people and bride-groom side. Here are quotations given as follows:

I think, mingling with patients can cause problems in my physique. (An adolescent male participant from FGD)

If someone visits home of a patient, they can be contaminated. (Adolescent male participant from FGD)

It becomes impossible to arrange a marriage for female patients. It is said that new-born of that female will also be mentally sick. (Adult female respondent from FGD)

People treated autism and disabilities as mental illness. An adult participant from FGD with PWD said, “disability and madness are considered the same in our country. I witnessed that someone has no hand, people called that person mad. Again, someone has impairment in their legs due to polio or other reasons, but people labeled them as insane people.” Any kinds of mental illness meant insane people for them. Whenever people with psychological problems came to their mind, they assumed that people could not maintain personal hygiene. They also assumed patients as bad people and as attention seekers. An adolescent patient said, “they said like this- o depressed. Now this girl wants attention, or this boy wants attention.”

In addition, people considered patients as incompetent in terms of skills in the workplace. They were not qualified and skilled enough to execute any sort of job. Colleagues and office authorities believed that patients could not follow office protocol and could not lead social and family life.

Furthermore, people viewed mental illness as not an illness and it could be cured by itself. An adolescent female patient said, “yes, yes. They do not accept mental illness; they think that it is an excuse.” They also alleged that psychological problems were not for male. They believed that marriage could cure psychological problems. An adult male patient shared his experience. He said, “family members are repeatedly stating one thing that is marriage. Patient maybe get well soon after getting married.”

Family would lose its social status if someone had mental illness. One respondent from a FGD described how mental illness became the cause of losing family pride.

My social status will be diminished due to my son’s psychological condition. I won’t take my son outside of my family. If I take him out, other people will notice him and call him as a mad. (Female participant from FGD with female teacher)

#### Discrimination toward patients

Patients experienced discrimination from their family members. Family members were afraid of losing family pride; therefore, they did not allow patients to go outside of the house. They even kept patient captive during family occasions. A counseling psychologist shared his experience regarding the confinement of a patient. He said, “patients are kept confined into a room.”

Family members did not offer enough support and care to the patients, likely the care they provided to their other members having a physical illness. In addition, patients were deprived of getting family property. Divorce was a very common consequence for both spouses. In other cases, it was found that family members expelled patients from home. They beat patients.

An adult male patient said, “the family having a member with psychological problems, villagers try to understand them that it is the consequence of their sin.” Society neglected patients and treated their family as inferior. Expelling patients’ families from rental houses or society was a very common phenomenon. In addition, patients were not invited to any social occasions. If they went to join festivals, people did not mingle with them. Moreover, people also witnessed that community members beat patients.

### Theme 3: professional stigma

The third theme emerged from the narratives of all categories of respondents. Prejudice and discriminatory attitude of healthcare professionals (HP) and general people toward patients and MHPs were the main concern for this theme. Explanation of three categories of the professional theme is given below:

#### From other HPs to patients

The other HPs considered patients as insane, attention seekers, aggressive, and inferior. An adolescent male participant from FGD with students commented that a responsible physician treated patients as mad. Other members of this group supported this notion. A general physician said, “so, in that case, I can say that she is an attention seeker. I mean, they want attention due to, maybe, they are stressed. They are so stressed that we literally did not find any physical problems.”

Furthermore, HP neglected psychological problems. They suggested that through marriage mental illness would be cured. An adult female participant said, “doctor listened everything and told that give marriage to your girl, everything will be alright. This is very pathetic.” In addition, misbehavior with patients in government hospitals was frequently shared information from participants of different focus group discussions and in-depth interviews.

#### From general people and patients to mental HP (MHP)

General people including patients held a stigmatizing attitude toward MHP. For instance, an adult female respondent from FGD with working women said, “there is a custom in society that who study psychology are mad. They are becoming a complete insane.” A psychosocial counselor shared his experience about people’s conception against MHP. He said, “my colleagues thought that since I am a doctor for insane, I have also psychological problems. So, it should not be mingled with me.”

Furthermore, people assumed that if they consulted an MHP like psychiatrist or psychologist, they would become insane. In addition, people believed that psychiatrists prescribed sleeping pills frequently. An adult female participant from FGD said, “too many sleeping pills cause him to fall asleep. People or family members think that my child is fine.” Moreover, mental health treatment centers held stigma by itself. An MHP said that if neighbors saw someone visited a mental health treatment center, they treated that family as a crazy family because of visiting or consulting MHPs. People also assumed that the staffs of mental health centers were unfriendly.

#### From other HP to MHP

A psychiatrist shared physicians from other departments devalued psychiatry profession and psychiatrists. He commented that they also treated patients differently. He said, “they thought that psychiatrist does not understand anything. They are doctor of insane people.” Moreover, General physicians offered psychiatrists a label as doctor of insane. A general physician said, “psychiatrists are called of doctor of insane.”

### Theme 4: institutional stigma

Health professionals and general people shared their experiences of discriminatory behavior toward patients. Consciously or unconsciously a culture of discrimination and prejudice against mental illness were developed and practiced in the different organizations.

#### Culture of negative attitude and belief

An adolescent female respondent from FGD said, “when a student having mental illness answers incorrectly to the questions asked by their teachers or classmates, she must not be kept in the institution. If she goes to another institution, they will not give her a chance after checking the records from the old institution. There is no place for her.” They had a lack of opportunity for education in comparison to other students. An adult participant from FGD with PWD said, “school authority does not want to admit them.” Most importantly, they were restricted in attending school events. In addition, some teachers and parents of other children did not accept them warmly. Most of their class friends teased them. One respondent explained how class friends teased patients.

Before considering his capabilities, people said, “what does he know?” As a result, these people lessen their confidence level. Then, they become more mentally ill person. For example, we have a classmate and friend who is sort of foolish and psychologically weak. Other students start to laugh when that friend talks. Because that friend cannot talk in an organized manner. (Adult male respondent from FGD)

A culture of negative attitude and belief system were formed in the educational settings. This culture was practiced not only in educational settings, but also in the workplace. For example, higher authority of the organizations believed that patients could not be able to lead a normal life; therefore, they were reluctant to keep patients at their workplace. An HP said, “well, here I share an experience. One of my well-known uncles was a Major in the army. He was a very good person. However, he was forced to retire after having a mental illness.” Getting a job was also very challenging for patients because of this culture of negative attitude and belief against patients. They received less salary and other facilities in comparison to other staff. An adult participant from FGD with transgender group said, “we were treated as person with gender disability. We did not get same salary as other staff of the organization.”

#### Policies of organizations

Intentionally or unintentionally psychiatry department faced discrimination in comparison to other health departments. A general physician said, “other departments such as medicine, surgery, gynecology, ENT are very big departments. But psychiatry department is very small.” In addition, an inadequate budget for conducting training and research was allocated for psychiatry departments.

General physicians had a gap in knowledge in psychiatry. They did not receive (or received less) training in psychiatry after their Bachelor of Medicine and Bachelor of Surgery (MBBS) degree. Even, MBBS curriculum did not emphasize psychiatry education. Here is a quotation from a psychiatrist:

We should put more emphasis on psychiatric education in our MBBS curriculum. For instance, there is no distinguished examination system for psychiatry. There is no additional viva board for psychiatry. Here, in viva there are questions for five to ten points. ….It is observed that someone does not know psychiatry after being a doctor. (Psychiatrist)

## Discussion

Most of the research on stigma against mental illness emphasized on impact of stigma on treatment-seeking behavior. The findings of this study have significant contributions to the literature in the context of Bangladesh as well as in the similar culture of other countries specially the Southeast Asian countries because this study has explored the stigma according to the types of stigma. It has also explored the impact of corresponding stigma in the context of Bangladeshi culture.

### Self-stigma

Participants of this study (i.e., patients’ category of sample) have internalized shame against themselves because of having a mental illness (Thornicroft et al., 2022). Self-stigma would develop when patients would believe the prejudices about mental illness against themselves and started to apply on them (Yanos et al., 2015; Thornicroft et al., 2022). Participants of this study have prejudice about the lifestyle and physical appearance of patients. For example, they assume that the lifestyle of patients having a mental illness will be messy and they will wear dirty dresses. They learn these forms of prejudices from their surroundings people (e.g., family, friends, community members, teachers, HPs, and relatives). They do not only learn it, but also experience the discriminatory attitude toward them. Hence, some assumptions about people with mental illness has already in their thought. In addition, several studies provided evidence that patients experienced self-stigma because of having a mental illness (Brohan et al., 2010; West et al., 2011).

Participants develop a fear of not getting married because of their mental illness. Previous studies have supported this finding. For example, the probability of getting married becomes poor after having a serious mental illness (Hailemariam et al., 2019). Patients even experience challenges such as finding intimate partners. Female patients experience more challenges than men in terms of getting married and continuation of marital life, even after recovery from their psychological problems (Hailemariam et al., 2019). For married female patients, they also encounter gender-based violence (Afe et al., 2016).

Respondents share that their family members are ashamed of having a patient in their family. They also report that their family members cannot present them to society. Subu et al. (2021) found that family felt ashamed when community people knew that they had a family member with a psychological problem. In another study it was found that family could be ashamed because of having a relative with a mental illness (Corrigan et al., 2016). Hence, this finding has aligned with prior research findings.

Interpersonal relationships are hampered because of stigma (Wong et al., 2009; Boyd et al., 2010) where ignorance becomes a common phenomenon (Corrigan et al., 2006; Ahmedani, 2011). Participants of this study also experience ignorance and avoidance from their neighbors during social gatherings. Moreover, society considers them dangerous. Minas and Diatri (2008) found that community people treat patients as violent.

Another finding is that discrimination toward self whereas patients perform discriminatory behavior toward themselves. Yanos et al. (2015) found that the expected treatment outcome did not yield because of self-stigma. In addition, poor self-efficacy (Kleim et al., 2008; Livingston, 2013), diminished self-esteem (Link et al., 2001; Corrigan, 2004; Livingston, 2013), and hopelessness (Livingston, 2013) are significant outcome of self-stigma that inhibit patients to respond against treatment. Consequently, their self-care system starts to decrease.

### Public stigma

Erroneous belief, prejudice, and fear against mental illness and patients are responsible for the development of public stigma (Ahmedani, 2011; Corrigan et al., 2012). Participants of this study report that they are afraid of both the patients and mental illness. For example, they have a belief of manifestation of mental illness is that mental illness is caused because of black magic, bad spirit, or supernatural powers. Even healthcare professionals such as general physicians have been influenced by misconceptions and fear (e.g., patients as violent). As a general people of the community, they have this traditional understanding of manifestation of public stigma. Lack of conscious awareness (Marková, 2016) can play an important role in having this understanding for physicians. However, people perceive mental illness as contagious that has a tremendous impact on both patients and family members. Literature reviews suggest that mental illness is also considered as contagious (Rose, 1998; Bilić and Georgaca, 2007; Walsh and Foster, 2020). Respondents of this study do not mingle with patients, and they avoid visiting patients’ houses because of the fear of contamination. In addition, getting married to female patients becomes impossible. The understanding of the form of contamination and marriage of female patients vary culture to culture. For example, female patients of low- and middle-income countries such as Ethiopia experience the challenge of getting married (Hailemariam et al., 2019). Bangladesh is not excluded from this list.

Research evidence suggests that autism is treated as psychological problem (Soron, 2015) whereas participants of this study also share that autism and disabilities are considered as mental illness. In addition, one of the important findings of this study is that men perceive that mental illness is not for them. A plausible explanation is the effect of the “ideas of masculinity” (Ogrodniczuk et al., 2016) in Bangladeshi society. Cultural norm teaches men that they are strong, and people consider them weak if they cry or express emotions. For instance, an adolescent female respondent shared her opinion against the question of why mental illness is not for men. She said, “girls can share their feelings, but boys cannot. If a boy cries, other people ask why you are crying being a boy. It does not look good.” Moreover, men are taught to be successful and providers for their family (Oliffe et al., 2013). The manhood and their role in the family and society may prohibit addressing their psychological problems and emotions.

Furthermore, family and society consider marriage as a cure to mental illness. Data analysis indicates that family members, society, and general physicians refer “marriage” as a cure for the mental illness. Hence, there will be an overlap. A possible explanation is that HPs are members of society, and they learn this prejudice from society that marriage will cure psychological problems.

However, research evidence suggests that patients experience discrimination due to public stigma (Corker et al., 2013) that affects their life significantly (Martin et al., 2000). Subu et al. (2021) found that community people committed violent behavior toward patients. The findings of the present study indicate that family and society members beat patients. Moreover, family members do not allow patients to visit outside because of a fear of losing family pride and negative criticism from society members. Consequently, patients are enclosed in a room. This finding is consistent with the findings of Subu et al. (2021). Furthermore, both family and patients are affected due to impact of social stigma. We have found that society neglects both family and patients. They encounter challenges to rent an apartment.

### Professional stigma

Professional stigma affects the patients’ well-being and treatment process (De Hert et al., 2011). It is also considered as one of the prime barriers to getting standard physical treatment for patients (Thornicroft et al., 2007; Henderson et al., 2014; Knaak et al., 2015). Several studies found that patients felt devalued, dehumanized, and rejected by HPs (Clarke et al., 2007; Barney et al., 2009; Thornicroft et al., 2010; Hamilton et al., 2016). The respondents of this study also share that HPs except MHPs treat them as insane, attention seekers, and violent. In addition, they are victims of misbehavior from the staff and general physicians from government hospitals. Research evidence suggests that physicians provided consultancy to patients with a demeaning manner (Clarke et al., 2007; Barney et al., 2009; Thornicroft et al., 2010; Hamilton et al., 2016). Moreover, we have found that general physicians neglect mental illness and give advice patients to get married as a cure of their psychological problems.

General people and patients have prejudices toward MHPs and treatment centers. For example, consulting an MHP offers patients a label with different offensive terms such as “mad” (National Institute of Mental Health, 2021). The findings of this investigation also align with this research evidence. In addition, patients and general people have a fear of getting prescription of sleeping pill from psychiatrists. They also experience unfriendly behavior from staff of mental health treatment centers.

Psychiatric nurses are confronted with discriminatory attitude from non-mental nurses because of working in mental health hospitals. Other people also call them and their patients as “crazy” (Subu et al., 2021). Mental health professionals such as psychiatrists of this inquiry report that doctors from non-mental health departments call them doctor of insane. They also demean the psychiatry profession, patients, and psychiatrists.

### Institutional stigma

Due to institutional stigma, patients are deprived of different opportunities from their workplace, and academic institutions (Thornicroft et al., 2022). The findings of the present study are coherent with other studies. For example, recruiters and employers have prejudices and discriminatory attitudes against patients in Indonesia till now (Subu et al., 2021) that is consistent with the findings of our study. In Bangladesh, a culture of negative attitude and belief has been embedded in the workplace and educational settings whereas getting and keeping a current job become very challenging for patients. Several studies found that patients encountered challenges in getting a job (Cook, 2006; Brohan et al., 2010; Cechnicki et al., 2011), and keeping the current job (Stuart, 2004). In addition, patients receive less employment benefits or even salary than other colleagues. Stuart (2004) also found that employers provided less benefits to the patients.

Bangladesh is a signatory country of inclusive education (IE) where it will ensure participation and access to education for all students (UNESCO, 1994). The country has also reflected the philosophy of IE in its law (Ministry of Law Justice and Parliamentary Affairs, 2019) where the law ensures participation and access education for all students irrespective of disability including students having a mental illness. The findings of this study contradict with the implementation of IE and laws in Bangladesh. For example, respondents share that patients cannot participate or get full access to educational institutions because of having psychological problems.

The government of Bangladesh allocates only 0.44% of its total budget to health care whereas even 0.11% people of the entire population do not get free psychiatric medication (Alam et al., 2021). It becomes impossible to organize training and research activities for psychiatry departments with this deficient budget. In addition, the country has only four beds per 10,000 people in mental health hospitals (Islam and Biswas, 2015). Moreover, inadequate MHPs along with insufficient fund and facilities, and stigma are responsible for not implementing the mental health policies in Bangladesh (Islam and Biswas, 2015). There is a dearth of literature against update status of the implementation of the mental health policy for Bangladesh.

## Strengths and limitations

The present study has various strengths and limitations. We have included diverse participants that represent the entire community of the country. For example, school and college-going students, recovered substance users, students from religious academic institutions, job holders, people from indigenous community, persons with disabilities, transgender community members, adolescent with a single parent or parentless group, housewives, patients, and healthcare professionals. Exploration of the lived experiences from marginalized population (e.g., substance users, indigenous community, PWD, and transgender community) has added an additional dimension to the findings. In addition, we have collected data covering all divisions of the country.

The data contains individuals’ thoughts, experiences, and opinions from participants’ perspectives. It is difficult to recruit research respondents with the same knowledge and understanding of the research contexts. Furthermore, institutional stigma covers a wide range. Exploration of institutional stigma from different organizations such as policies of the organization that limit the opportunity for patients is beyond the time and allocated resources for this study. Therefore, exploration of institutional stigma in terms of organizational policies is considered a limitation for this study. In addition, the research findings with different groups of respondents from different cultural and income levels of countries may yield new insights. Considering this notion, generalization for other cultural contexts will be another limitation of the study.

## Conclusion

This study reveals a deep understanding about stigma against mental illness in the context of Bangladesh. Though it has some limitations, the findings will help MHPs to tailor therapeutic interventions for female, marginalized community such as substance users, people from lower cast and socioeconomic status, PWD, Indigenous community, and transgender community. Because individuals from these communities have already encountered different discrimination; discrimination against them can be increased significantly after having a mental illness. Moreover, MHPs can be aware of the impact of stigma; therefore, they can address it in their treatment effectively. In addition, HPs from other health departments can change their conception against mental illness, patients, and MHPs positively. Finally, policymakers can develop an effective healthcare service in the country by addressing the findings of this study.

## Supporting information

Roy and Chowdhury supplementary material 1 Roy and Chowdhury supplementary material

Roy and Chowdhury supplementary material 2 Roy and Chowdhury supplementary material

Roy and Chowdhury supplementary material 3 Roy and Chowdhury supplementary material

Roy and Chowdhury supplementary material 4 Roy and Chowdhury supplementary material

## Data Availability

The transcription can be shared upon request to the corresponding author.

## Supplementary material

The supplementary material for this article can be found at http://doi.org/10.1017/gmh.2024.107.
