# Supplementary material for: Exploring the stigma against people with mental illness in Bangladesh
Source: Glob Ment Health (Camb). 2024 Nov 11;11:e108. doi: 10.1017/gmh.2024.107 (PMC11704370; doi:10.1017/gmh.2024.107)
Supplement: Roy and Chowdhury supplementary material 1 — Roy and Chowdhury supplementary material [file S2054425124001079sup001.docx]

Table 1. Characteristics of patients

| Participants | Age | Sex | Division | Education | Diagnosis/  symptoms | Profession | Socio Economic Status | Marital status | # of Family members | Religion |
| --- | --- | --- | --- | --- | --- | --- | --- | --- | --- | --- |
| 1 | 25 | Male | Dhaka | SSC^1^ | OCD (F-42) | Banker | Middle class | Single | 4 | Hindu |
| 2 | 28 | Female | Dhaka | MA^2^ | Anxiety and Personality issue | Unemployed | Middle | Single | 5 | Islam |
| 3 | 40 | Female | Rangpur | Non-literate | OCD | Housewife | Lower Middle | Married | 6 | Islam |
| 4 | 17 | Male | Rangpur | HSC^3^ | Anxiety disorder | Student | Middle | Single | 4 | Hindu |
| 5 | 17 | Male | Chattogram | Class 12 | Mood disorder | Student | Middle class | Single | 3 | Islam |
| 6 | 48 | Female | Chattogram | MS^4^ | Trauma and Stressor related disorder | Teacher | Middle class | Married | 3 | Islam |
| 7 | 17 | Female | Khulna | HSC | Mild Depression | Student | Middle class | Single | 4 | Hindu |
| 8 | 34 | Male | Khulna | Masters | Anxiety disorder | Businessman | Middle class | Married | 6 | Islam |
| 9 | 17 | Female | Rajshahi | HSC (2nd year) | Panic disorder | Student | Middle class | Single | 4 | Islam |
| 10 | 22 | Male | Rajshahi | Hons (1st year) | BPD with Depression | Student | Middle class | Single | 3 | Islam |
| 11 | 18 | Female | Sylhet | HSC | Depression | Student | Middle class | Single | 2 | Islam |
| 12 | 17 | Male | Sylhet | HSC | Anxiety | Student | Middle class | Single | 3 | Islam |
| 13 | 14 | Male | Mymensingh | Class 7 | Repetitive behaviour, low mood, loneliness | Student | Lower Middle Class | Single | 4 | Islam |
| 14 | 24 | Female | Mymensingh | BDS^5^ (4th year) | Low mood, poor social interaction | Student | Middle class | Single | 5 | Islam |

Note: 1-Secondary School Certificate, 2-Master of Arts, 3-Higher Secondary Certificate, 4-Master of Science, 5-Bachelor of Dental Surgery
