# Supplementary material for: Exploring the stigma against people with mental illness in Bangladesh
Source: Glob Ment Health (Camb). 2024 Nov 11;11:e108. doi: 10.1017/gmh.2024.107 (PMC11704370; doi:10.1017/gmh.2024.107)
Supplement: Roy and Chowdhury supplementary material 2 — Roy and Chowdhury supplementary material [file S2054425124001079sup002.docx]

Table 2. Characteristics of healthcare professionals

| Participants | Age | Sex | Division | Education | Profession | Socio Economic Status | Marital status | # of Family members |  | Religion |
| --- | --- | --- | --- | --- | --- | --- | --- | --- | --- | --- |
| 1 | 31 | Male | Dhaka | MS^1^ | Psychologist | Higher middle class | Married | 5 |  | Hindu |
| 2 | 43 | Male | Rangpur | MBBS^2^, MD^3^ | Psychiatrist | Middle class | Married | 4 |  | Islam |
| 3 | 31 | Female | Barishal | FCPS^4^, part-1 | Doctor | Middle class | Married | 6 |  | Islam |
| 4 | 48 | Male | Dhaka | MD, Psychiatry | Psychiatrist | Higher middle class | Married | 4 |  | Islam |
| 5 | 32 | Male | Chattogram | MBBS | Doctor | Middle class | Married | 3 |  | Hindu |
| 6 | 35 | Male | Khulna | Masters | Village doctor | Middle class | Married | 3 |  | Hindu |
| 7 | 35 | Female | Rajshahi | BSc^5^ in Nursing | Nurse | Middle class | Married | 2 |  | Christian |
| 8 | 29 | Male | Sylhet | MS | Psychosocial counsellor | Middle class | Single | 6 |  | Islam |
| 9 | 40 | Female | Mymensingh | MD, Paediatric | Child health physician | Higher middle class | Married | 4 |  | Islam |

Note: 1-Master of Science, 2-Bachelor of Medicine and Bachelor of Surgery, 3-Doctor of Medicine, 4-Fellowship of the College of Bangladesh and Surgeons, 5-Bachelor of Science
