# Supplementary material for: Exploring the stigma against people with mental illness in Bangladesh
Source: Glob Ment Health (Camb). 2024 Nov 11;11:e108. doi: 10.1017/gmh.2024.107 (PMC11704370; doi:10.1017/gmh.2024.107)
Supplement: Roy and Chowdhury supplementary material 3 — Roy and Chowdhury supplementary material [file S2054425124001079sup003.docx]

Table 3. Characteristics of participants for FGD

| Division | FGD with people without mental illness | | | | | Total  Groups | Number of participants |
| --- | --- | --- | --- | --- | --- | --- | --- |
|  | Adolescent | | Adult | | |  |  |
|  | Male | Female | Male | Female | LGBT |  |  |
| Barishal |  | Student | Community | Student |  | 3 | 18 |
| Chattogram | Student | Student | Indigenous | Community (mixed Bengali and Indigenous) |  | 4 | 18 |
| Dhaka | Substance abuse (Recovered) | Student | Community, and  PWD (3 male & 2 female) | Working- women | Transgender | 6 | 33 |
| Khulna | Having single parent/parentless adolescents | Having single parent/parentless adolescents | Police | Working- women |  | 4 | 26 |
| Rajshahi | Student | Student | Community | Working- women |  | 4 | 24 |
| Rangpur | Student | Student | Community | Teachers |  | 4 | 24 |
| Mymensingh | Community | Student | Community | Housewife |  | 4 | 25 |
| Sylhet | Substance abuse | Madrasa student | Community | Indigenous |  | 4 | 25 |
| Total | 7 | 8 | 9 | 8 | 1 | 33 | 193 |
