# Supplementary material for: Exploring the stigma against people with mental illness in Bangladesh
Source: Glob Ment Health (Camb). 2024 Nov 11;11:e108. doi: 10.1017/gmh.2024.107 (PMC11704370; doi:10.1017/gmh.2024.107)
Supplement: Roy and Chowdhury supplementary material 4 — Roy and Chowdhury supplementary material [file S2054425124001079sup004.docx]

Table 4. Themes and categories

| Themes | Categories |
| --- | --- |
| Theme 1: Self-stigma | Internalized negative attitudeDiscrimination toward self |
| Theme 2: Public stigma | - 1. Prejudice against patients   2. Discrimination toward patients |
| Theme 3: Professional stigma | - 1. From other health professionals (HP) to patients  From general people and patients to Mental HP (MHP)From other HP to MHP |
| Theme 4: Institutional stigma | - 1. Culture of negative attitude and belief   2. Policies of organizations |
