# Supplementary material for: Exploring the stigma against people with mental illness in Bangladesh
Source: Glob Ment Health (Camb). 2024 Nov 11;11:e108. doi: 10.1017/gmh.2024.107 (PMC11704370; doi:10.1017/gmh.2024.107)
Supplement: Peer review history

# Editor report: Recommendation: Exploring the stigma against people with mental illness in Bangladesh — R0/PR2

**DOI:** 10.1017/gmh.2024.107.pr2

**Affiliations:** The George Institute for Global Health India, India

Please go through the comments made by the reviewers which are quite exhaustive and address them point wise.

The methods and interpretation of results need to be carefully reassessed and drafted. the discussion is particularly weak and needs significant restructuring and modifying to address relevant literature from South Asia where there is a significant body of work on stigma and discrimination. How are these results similar or dissimilar to those?

# Editor report: Decision: Exploring the stigma against people with mental illness in Bangladesh — R0/PR3

**DOI:** 10.1017/gmh.2024.107.pr3

**Affiliations:** London School of Hygiene & Tropical Medicine, United Kingdom of Great Britain and Northern Ireland

No accompanying comment.

# Editor report: Recommendation: Exploring the stigma against people with mental illness in Bangladesh — R1/PR5

**DOI:** 10.1017/gmh.2024.107.pr5

**Affiliations:** The George Institute for Global Health India, India

Please respond to the authors comments.

I also do not see any reference to the Lancet Commission on Stigma and Discrimination. Also there is considerable literature from India and some from Pakistan too which should be included either int he introduction or discussion. From India especially there are key research which has formally evaluated stigma over time and also longitudinally. As the authors claim that literature is limited from LMIC, this is particularly needed to indicate how extant research in other neighbouring countries are different from the current research. Otherwise that statement is inaccurate. The introduction and discussion section should clearly articulate that.

# Editor report: Decision: Exploring the stigma against people with mental illness in Bangladesh — R1/PR6

**DOI:** 10.1017/gmh.2024.107.pr6

**Affiliations:** London School of Hygiene & Tropical Medicine, United Kingdom of Great Britain and Northern Ireland

No accompanying comment.

# Editor report: Recommendation: Exploring the stigma against people with mental illness in Bangladesh — R2/PR8

**DOI:** 10.1017/gmh.2024.107.pr8

**Affiliations:** London School of Hygiene & Tropical Medicine, United Kingdom of Great Britain and Northern Ireland

No accompanying comment.

# Editor report: Decision: Exploring the stigma against people with mental illness in Bangladesh — R2/PR9

**DOI:** 10.1017/gmh.2024.107.pr9

**Affiliations:** London School of Hygiene & Tropical Medicine, United Kingdom of Great Britain and Northern Ireland

No accompanying comment.

# Editor report: Recommendation: Exploring the stigma against people with mental illness in Bangladesh — R3/PR11

**DOI:** 10.1017/gmh.2024.107.pr11

**Affiliations:** London School of Hygiene & Tropical Medicine, United Kingdom of Great Britain and Northern Ireland

No accompanying comment.

# Editor report: Decision: Exploring the stigma against people with mental illness in Bangladesh — R3/PR12

**DOI:** 10.1017/gmh.2024.107.pr12

**Affiliations:** London School of Hygiene & Tropical Medicine, United Kingdom of Great Britain and Northern Ireland

No accompanying comment.
